# Behavioral Alterations in Male Zebrafish After Administration of Androgen Receptor Blockers and an Activator

**DOI:** 10.3390/biology15050393

**Published:** 2026-02-27

**Authors:** Ching-Yu Huang, Gilbert Audira, Ross D. Vasquez, Honeymae C. Alos, Hung-Yu Lin, Chung-Der Hsiao, Chih-Hsin Hung

**Affiliations:** 1 Institute of Biotechnology and Chemical Engineering, I-Shou University, Kaohsiung 840203, Taiwan; ed102747@edah.org.tw; 2 Division of Urology, Department of Surgery, E-Da Dachang Hospital, Kaohsiung 80794, Taiwan; 3 Department of Bioscience Technology, Chung Yuan Christian University, Chung-Li 320314, Taiwan; gilbertaudira@yahoo.com; 4 Department of Pharmacy, Faculty of Pharmacy, University of Santo Tomas, Manila 1015, Philippines; rdvasquez@ust.edu.ph; 5 Research Center for the Natural and Applied Sciences, University of Santo Tomas, Manila 1015, Philippines; honeymaealosgs@ust.edu.ph; 6 The Graduate School, University of Santo Tomas, Manila 1015, Philippines; 7 School of Medicine, College of Medicine, I-Shou University, Kaohsiung 84001, Taiwan; ed100464@edah.org.tw; 8 Research Center for Aquatic Toxicology and Pharmacology, Chung Yuan Christian University, Taoyuan City 320314, Taiwan

**Keywords:** androgen, enzalutamide, apalutamide, dihydrotestosterone, zebrafish, behavior

## Abstract

Androgen receptor (AR) signaling is crucial for mediating male-typical behaviors across vertebrates. Enzalutamide (ENZ) and apalutamide (APA) are two second-generation androgen receptor inhibitors (SGARIs) that have been primarily used in the treatment of prostate cancer. However, these drugs still possess side effects, and there remains limited information regarding their behavioral and neurophysiological impacts following chronic exposure in non-mammalian animal models, particularly in fish. Thus, this study aimed to evaluate the behavioral alterations in adult male zebrafish (*Danio rerio*) following exposure to specific AR blockers (ENZ and APA) and an activator, dihydrotestosterone (DHT), to provide a comprehensive comparison between each tested drug. Adult male zebrafish were exposed via aqueous immersion to each compound at a 1 ppm concentration for ~2 weeks and were subjected to a battery of behavioral tests. From the results, both AR blockers were found to slightly compromise fish locomotion, with contrary results observed in DHT-treated fish, which displayed an increased locomotor activity together with slight alterations in fish exploratory behaviors. Furthermore, ENZ also caused a tightened shoal formation in zebrafish, while exposure to APA was observed to slightly diminish the fear response of fish. On the other hand, the DHT-treated group displayed a higher level of aggression compared to the vehicle control group. In conclusion, functional modulation of androgen receptor signaling leads to significant alterations in male zebrafish behavior, particularly affecting fear responses, aggression, and anxiety-related behaviors. We believe that these findings could contribute to a deeper understanding of the relationship between androgens and behaviors in vertebrates, especially zebrafish.

## Simple Summary

Male hormones play an important role in shaping animal behavior, including movement, social interaction, fear, and aggression, and are governed by the androgen receptor. Certain drugs used to treat human prostate cancer work by blocking these receptors, and unfortunately, they may enter aquatic environments through wastewater, where they could affect fish. In this study, we examined how long-term exposure to two hormone-blocking drugs, enzalutamide and apalutamide, and one hormone activator, dihydrotestosterone, influenced the behavior of adult male zebrafish. Fish were exposed to each substance for about two weeks and then tested for changes in swimming activity, social grouping, fear responses, and aggression. The hormone-blocking drugs slightly reduced swimming activity and altered social or fear-related behaviors, while the hormone activator increased activity and aggression and changed exploratory behavior. These results show that disrupting male hormone signaling can strongly influence fish behavior in different ways. This research helps improve our understanding of how hormone-related chemicals affect animals and highlights potential risks to aquatic wildlife when such substances enter natural water systems. Additionally, it confirms that zebrafish are an effective model for studying how hormones control behavior, which helps scientists better understand the link between hormones and the brain in all backboned animals, including humans.

## 1. Introduction

Androgen, or male sex hormone, is a substance that belongs to a class of steroid hormones capable of developing and maintaining masculine characteristics in reproductive tissues, including male secondary sexual characteristics and reproductive behaviors, and contributing to the anabolic status of somatic tissues [1]. Androgen produces its effects by binding to androgen receptors (ARs), which belong to the steroid hormone receptor superfamily and modulate the transcription of specific genes [2,3]. The androgen–AR complex can either induce or suppress the androgen-responsive genes via binding to androgen-response elements that are generally located in the 5′ flanking region of androgen target genes [3]. The AR signaling axis has been a focus for the treatment of advanced prostate cancer, since ARs are necessary for the function, survival, and differentiation of prostatic tissue in the normal prostate. However, during carcinogenesis, the function of AR signaling is altered from tumor-suppressive to tumor-promoting [4,5,6,7,8]. Therefore, at present, the blockade of AR signaling to disrupt androgen signaling pathways remains a focus of the current clinical paradigm for the treatment of cancer [9].

Enzalutamide (ENZ) and apalutamide (APA) are two second-generation androgen receptor inhibitors (SGARIs) that block the AR signaling pathway and have been proven to improve survival outcomes in prostate cancer patients. Different from antiandrogens, these drugs do not have agonist effects in AR signaling but competitively inhibit androgen binding to the AR, thereby preventing the nuclear translocation of androgens and interactions with DNA, inducing apoptosis and decreasing tumor volume and the proliferation of cancer cells [10,11]. Besides their slightly different chemical structures, ENZ and APA are also approved for the treatment of different prostate cancers. While ENZ is approved for the treatment of patients with castration-resistant prostate cancer (CRPC), APA is approved for the treatment of patients with nonmetastatic CRPC (nmCRPC) [12]. Moreover, while APA acts with similar mechanisms, it binds with greater affinity to ARs compared to ENZ, which was supported by previous in vivo experiments that suggested that APA might have greater antitumor activity and lower penetration into the blood–brain barrier than ENZ [12,13,14]. Despite their benefit in the treatment of prostate cancer, these drugs still possess some side effects, including fatigue, dizziness, skin rash, and cardiotoxicity, which have been extensively studied in humans [12,15]. However, limited information is available regarding the behavioral and neurotoxic effects of these compounds in animal models, particularly under chronic exposure conditions, as summarized in Table S1 [16]. In mice, exposure to ENZ was demonstrated to reduce spontaneous activity and exploratory behavior associated with a decreased tyrosine hydroxylase (TH)-dopaminergic activity. Furthermore, while this drug did not substantially modify the spatial learning and memory performances of mice, its exposure led to a thigmotaxis behavior, impacting the cognitive score [17]. Moreover, in another prior study, ENZ was shown to induce bradycardia and death in zebrafish embryos in a dose-dependent manner [18]. Although pharmacokinetic characterization is essential for drug development in clinical contexts, behavioral studies in aquatic models primarily rely on exposure-based approaches to evaluate functional outcomes, including neurobehavioral alterations and endocrine disruption following chronic treatment [16].

Recently, zebrafish have emerged as an efficient animal model for assessing the neurotoxicity of various compounds, such as drugs and nanomaterials, due to their prominent properties, including genetic homology with most human genes and the conserved neurological systems that are comparable to mammals [19,20,21,22]. Moreover, besides their small sizes, low costs to breed, maintain, and transport, short generation times and life cycles, and high fertility rates, zebrafish also possess a large, robust, and variable behavioral repertoire, such as courtship behavior patterns, including mating behavior [23,24]. Thus, considering behavior as the most sensitive indicator of impact exposure, assessing zebrafish behaviors after chemical exposure could help in understanding how specific chemicals induce neurobehavioral and physiological changes at different levels [25].

**Figure 1:**
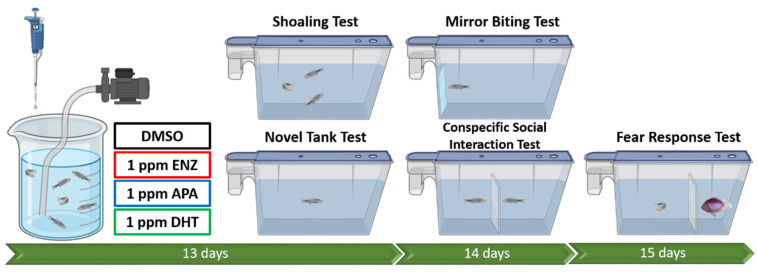
Experimental design regarding the evaluation of chronic exposure to AR blockers and activators at 1 ppm concentration in adult male zebrafish, as applied in the current study (created with biorender.com). In the fear response test, convict cichlid (*Amatitlania nigrofasciata*) was used as a visual stimulus to elicit the fear responses of the zebrafish.

The effects of androgens on secondary sexual characteristics and behavior are well described in vertebrates, with no exception for teleost fish, including zebrafish. In zebrafish, the AR is expressed in the developing brain during embryogenesis, while in adult fish, expression of the AR includes regions of the brain and testis [26,27]. Interestingly, a prior study strongly suggested that the zebrafish gene encodes the true orthologue of the human *ar* gene, with similarity in the lengths of the internal coding exons of *ar* genomic structures, suggesting potential conservation of ligand binding sites [26,28]. This feature makes zebrafish a powerful tool for investigating the link between androgen signaling and social behaviors. Thus, understanding how androgen receptor inhibitor drugs interact with zebrafish AR can provide insights into potential ecological risks associated with their environmental presence, considering that the impact of these drugs on non-target organisms is not fully understood. Therefore, the present study aimed to evaluate the behavioral effects of ENZ and APA in adult male zebrafish by conducting various behavior assays. In addition, the behavioral effects of dihydrotestosterone (DHT), an androgen receptor activator, were also evaluated to provide a comprehensive comparison between each tested drug. DHT is known as a potent androgen that possesses a higher affinity to AR than testosterone and acts peripherally by its stimulation of the male sex organs and spinal aspects of sexual reflexes [29,30]. In rats, there is evidence of DHT’s effect in enhancing masculine behavior when injected systemically or implanted directly into the central nervous system [30]. Unfortunately, this steroidogenic metabolite has only received a little attention in non-mammalian species, including fish. However, some prior studies have demonstrated that DHT is a potent androgen in fish; thus, it was intriguing to also evaluate the behavioral effects of this androgen in zebrafish [31]. In addition, molecular docking, a computational approach, would also be conducted to predict the binding energy between these drugs and the human endogenous AR. We hypothesized that male zebrafish treated with ENZ and APA would exhibit significant behavior alterations that might be related to the high binding affinity of these drugs to zebrafish AR, and these alterations would be different from those caused by DHT exposure. The overview of the current study is shown in Figure 1.

## 2. Materials and Methods

### 2.1. Fish Husbandry

Adult wild-type zebrafish were purchased from a local aquarium pet store and acclimated in the fish facility for at least 6 months. In the facility with a 14:10 h light/dark cycle, fish were housed in a glass tank (110 × 33 × 33 cm) filled with recirculating water that was filtered by UV light. The water temperature was maintained at 28.5 °C (pH 7.2–7.6) with water conductivity maintained between 300 and 1500 μS and ~7 ppm of dissolved oxygen. The fish were fed twice daily with lab-grown brine shrimp and commercial dry food (Taiwan Hung Kuo Industrial Co., Ltd., Taipei, Taiwan), each once daily. To maintain consistent nutritional status across groups, a ‘satiation feeding’ method was used, where approximately 3–4 pellets were provided per fish, and the volume of brine shrimp was adjusted to ensure total consumption within 1–2 min. This protocol prevented overfeeding and ensured that water quality parameters, particularly nitrogenous waste levels, remained stable. For the following experiment, a total of 120 healthy fish with a body weight and length ranging from 0.3 to 0.5 g and 2.5–3.5 cm, respectively, and normal morphology were used. The protocol for fish husbandry was based on the previous study [32]. The experiment was conducted according to the guidelines for the care and use of Laboratory Animals by Chung Yuan Christian University (CYCU) and approved by the Animal Ethics Committee of CYCU (IACUC Certificate Approval No. 112010 of 29 December 2022).

### 2.2. Chemical Exposures

Enzalutamide and apalutamide with 98% purity were purchased from Bidepharm (Shanghai, China). Meanwhile, dihydrotestosterone (DHT) and androstanolone, with a purity of 99.9%, were purchased from Tanmo Quality Inspection Technology Co., Ltd. (Changzhou, Jiangsu, China). Afterward, the chemicals were diluted to 10,000 ppm with DMSO. Next, by using a simple random allocation method to avoid experimental bias, the healthy adult male zebrafish from the holding tank were randomly divided into five groups: control vehicle with 1 ppm of DMSO and 1 ppm of ENZ, APA, and DHT [33]. Sex was determined based on established secondary sexual characteristics: males were identified by their slender, torpedo-shaped bodies and the presence of a golden-yellowish tint between the longitudinal stripes. Females, characterized by a more robust ventral region and silvery coloration, were excluded. To maintain a strictly male cohort, any individuals exhibiting ambiguous morphological traits were not used in the experiment. The exposure concentration of 1 ppm was selected to evaluate chronic neurobehavioral effects of androgen receptor modulation in adult zebrafish under sublethal conditions. In waterborne exposure systems, nominal concentrations do not directly correspond to internal tissue or brain levels due to uptake dynamics and metabolic processes. Therefore, concentrations are commonly selected based on their ability to induce behavioral alterations without causing overt toxicity. The chosen concentration was lower than those reported in prior zebrafish larvae and rodent studies and allowed for sustained exposure over 14 days [17,18]. Each group for each replication consisted of 10 fish and was reared in a glass beaker with 2 L of aerated exposure water for ~14 days. The exposure medium was refreshed every two days to maintain the drug concentration and prevent any possibility of bacterial infection caused by bad water quality, which might come from excess food after feeding. This experiment was conducted in three replications with a total of 30 fish for each group.

### 2.3. Zebrafish Behavior Assays

Here, a total of five types of zebrafish behavior assays, namely novel tank, shoaling, aggressiveness, conspecific social interaction, and fear response tests, were conducted on three consecutive days in a room with a 26 ± 1 °C temperature and minimum noise. When multiple behavioral tests were conducted on the same day, individual fish were allowed a resting period of approximately 4–5 h between tests before undergoing the subsequent assay. All of these behavior tests used a trapezoid plastic tank (15.9 cm along the diagonal side, 22 cm bottom, and 15.2 cm high) filled with ~1.25 L of filtered water. On the thirteenth day of exposure, the novel tank test was conducted, followed by the shoaling assay. In the novel tank test, the tested fish were individually moved to the test tank, and their behaviors were recorded for 1 min every 5 min for 31 min. Meanwhile, in the shoaling test, a group of fish with a shoal size of three fish was put in the test tank. After ~5 min of acclimation, their behaviors were recorded for 5 min. On the following day, aggressiveness and conspecific social interaction assays were conducted. During the aggressiveness test, the zebrafish’s behavioral responses to their reflection that was produced by the mirror that was inserted on one side of the test tank wall were recorded for 5 min after a ~1 min acclimation period. However, in the conspecific social interaction test, the mirror was replaced with a transparent separator that was put in the middle of the test tank to separate the tested fish from its conspecific. After a naïve female zebrafish, as its conspecific, was put in the test tank and allowed to acclimate with the environment for ~5 min, the behaviors of the tested fish were recorded for 5 min to observe its interaction with the conspecific female fish. Finally, on the fifteenth day of exposure, the fear response test, as the last behavior assay, was conducted. Similar to the conspecific social interaction test, this test also utilized the transparent separator; however, it was applied to separate the tested zebrafish from the freshwater convict cichlid (*Amatitlania nigrofasciata*) as a visual stimulus to elicit the fear responses of the tested fish, which were recorded for 5 min after ~1 min of acclimation. The applied behavior tests were according to the previous protocol [34]. All behavioral tests were conducted in a temperature-controlled room between 09:00 and 17:00 to minimize circadian influence. Behaviors were recorded using a Canon EOS D600 DSLR (Digital Single-Lens Reflex) camera (55–250 mm lens; Canon Inc., Tokyo, Japan) at 50 frames per second in a 1280 × 720 pixels video resolution. Afterward, the video files were processed using UMATracker software version 2019.0707.133407 to calculate various essential behavior endpoints [35]. Detailed information regarding the calculated behavior endpoints in each behavior test can be found in Table S2 [36]. The assays were done by the blind trained observers.

### 2.4. Statistical Analyses

All of the graphs and statistical tests were generated and conducted by GraphPad Prism (GraphPad Software version 8 Inc., La Jolla, CA, USA). Prior to the statistical tests, normality tests, including Kolmogorov–Smirnov, Shapiro–Wilk, D’Agostino–Pearson, and Anderson–Darling tests, were carried out to test the normality of data distribution. For the novel tank test, two-way ANOVA with Geisser–Greenhouse correction followed by Dunnett’s multiple comparisons test was used to calculate the significant differences between the treated and untreated groups during the whole 30 min test. Meanwhile, for other behavior tests, Kruskal–Wallis followed by Dunn’s multiple comparisons test, which was used to evaluate the statistical differences between control and treated groups, was used since the assumption of normal distribution was not required by this nonparametric test, considering the non-normal distribution of the data [37]. Asterisks of *, **, ***, or **** were used to mark the significant differences between treated and vehicle control groups if *p*-values were < 0.05, <0.01, <0.001, or <0.0001, respectively. In addition, prior to the experiment, a statistical power analysis was also conducted to determine the sample size that would be used in the current study, which later it was found that with a sample size of 30, data with a 99% confidence interval and a 4.5 margin of error could be obtained [38].

### 2.5. PCA and Heatmap Clustering Analysis

The mean value for each behavior endpoint (Table S2) was computed and stored in a comma-separated values (.csv) file using Microsoft Excel. This file was then uploaded to ClustVis (https://biit.cs.ut.ee/clustvis/ (accessed on 24 February 2026)), and unit variance scaling was applied to each row [39]. For the principal component analysis (PCA), Singular Value Decomposition (SVD) with the imputation method was used, as the dataset had no missing values. Finally, the PCA and heatmap results were exported to the system.

### 2.6. Molecular Docking

The in silico experiment was performed using the Androgen receptor in complex with tiratricol, through the use of reverse molecular docking. The AR protein (PDB: 2PIT) of *Homo sapiens* (Human) origin, with a resolution of 1.76 Å, was extracted from the RCSB protein database. The protein was prepared by removing molecules that may contribute to distortion, such as water molecules and calcium ions, with UCSF Chimera (version 1.19). On the other hand, ligands, which were ENZ, APA, DHT, and the native ligand tiratricol, were extracted from the PubChem database. Through the PyRx software (version 0.8), the minimization of ligands was done. Using the Vina wizard tool, reverse docking was done with a grid size as indicated in Table 1.

Reverse docking in reference to the native ligand amino acid interactions with PHE673, PRO723, ASN727, GLU837, TYR834, and ARG840. RMSD calculation or superimposition of the native ligand was performed, resulting in 0.591 Å.

## 3. Results

### 3.1. Chronic Exposure to AR Blockers and an Activator Affected the Locomotion and Exploratory Behaviors of Zebrafish in a Novel Environment

**Figure 2:**
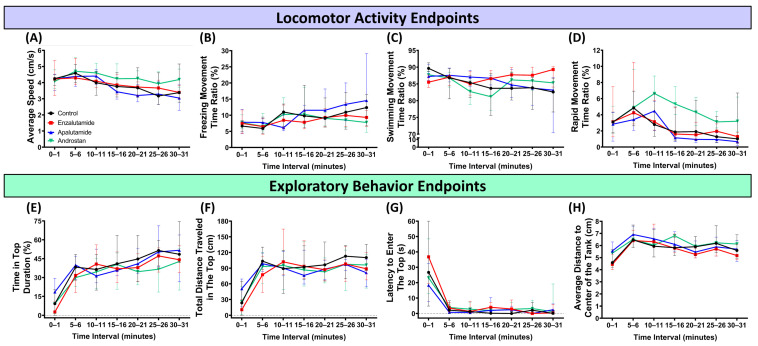
Locomotor activity ((**A**) average speed, (**B**) freezing time movement ratio, (**C**) swimming movement time ratio, and (**D**) rapid movement time ratio) and exploratory behavior ((**E**) time in top duration, (**F**) total distance traveled in the top, (**G**) latency to enter the top, and (**H**) average distance to center of the tank) endpoints of zebrafish after being systemically exposed either to 1 ppm of enzalutamide (ENZ) (red), apalutamide (APA) (blue), or dihydrotestosterone (DHT) (green), compared to vehicle control (black). The data are expressed as the median with an interquartile range. The statistical analyses were conducted by two-way ANOVA with Geisser–Greenhouse correction. To observe the treatment effect, Dunnett’s multiple comparison test was carried out. Detailed statistical results corresponding to Figure 2 are provided in Supplementary Table S3 (*n* = 30).

To evaluate the behavioral response of the fish toward a novel environment, a novel tank test was conducted. From the results, both compounds were found to be able to alter zebrafish behavioral response in a new environment, especially in terms of locomotion. Here, the most pronounced alterations in fish locomotion were displayed by the DHT-treated group, which showed statistically higher locomotion, as indicated by the greatly significant difference in average speed and significant difference in freezing movement time ratio compared to the control group (Figure 2A,B and Table S3). Meanwhile, slight differences were also observed in both AR-blocker-treated groups that were reflected in their freezing, swimming, and rapid movement time ratios; however, these changes were not potent enough to significantly change their overall average speed in comparison to the control group throughout the test (Figure 2A–D, Table S3). However, one has to keep in mind that after conducting a two-way ANOVA test, a significant *p*-value of interaction was found in all of the locomotion-related endpoints, indicating that the effect of the drugs differed depending on the time, thus reducing the significance of the calculated *p*-values by Dunnett’s multiple comparison test. Furthermore, in terms of the fish exploratory behaviors, while no statistical differences were observed in both AR blocker-treated groups, slight alterations were found in the DHT-treated group. This behavioral abnormality was indicated by the greatly significant difference in the time in the top duration than the untreated group, although no statistical differences were observed in the total distance traveled in the top and latency to enter the top portion of the tank (Figure 2E–G, Table S3). In addition, no obvious changes were found regarding fish horizontal position preference in all of the treated groups, as signified by the similar level of thigmotaxis to the control group (Figure 2H, Table S3). Nevertheless, these results indicated the potency of DHT in causing a hyperactivity-like behavior in zebrafish and alteration in exploratory behaviors of zebrafish, including the capability of both AR blockers in slightly disrupting fish locomotion during their habituation process in a novel environment.

### 3.2. Chronic Exposure to Dihydrotestosterone Enhanced the Aggression Level of Zebrafish

**Figure 3:**
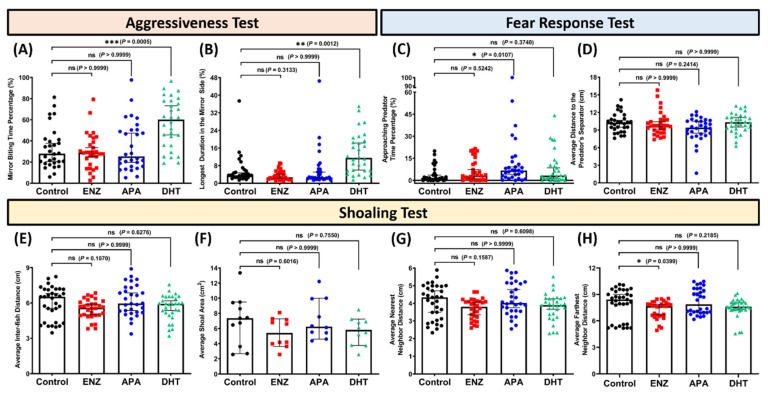
Mirror biting ((**A**) mirror biting time percentage and (**B**) longest duration in the mirror side)), fear response ((**C**) approaching predator time percentage and (**D**) average distance to the predator’s separator)), and shoaling ((**E**) average inter-fish distance, (**F**) average shoal area, (**G**) average nearest neighbor distance, and (**H**) average farthest neighbor distance)) behavior endpoints of zebrafish after being systemically exposed either to 1 ppm of enzalutamide (ENZ) (red), apalutamide (APA) (blue), or dihydrotestosterone (DHT) (green), compared to the vehicle control group (black). The data are expressed as the median with an interquartile range. The statistical analyses were conducted by the Kruskal–Wallis test followed by Dunn’s multiple comparisons test (*n* = 30, except *n* control in shoaling test = 33; ns = not statistically significant, * *p* < 0.05, ** *p* < 0.01, *** *p* < 0.001).

Next, one of the socially related behaviors of zebrafish was evaluated after chronic exposure to both AR blockers and DHT by conducting a mirror biting test, an established behavior assay to evaluate the fish’s aggressive behaviors. From the results, chronic waterborne exposure of both ENZ and APA did not statistically change the fish’s aggressiveness as shown in both behavior endpoints (Figure 3A,B). However, as expected, a substantially higher aggression level than the control group was displayed by the zebrafish after being chronically exposed to DHT, as indicated by the greatly significant differences in mirror biting time percentage and significant differences in longest duration on the mirror side compared to the untreated group (Figure 3A,B and Figure S2A). Furthermore, another social-related behavior assay, which was the conspecific social interaction test, was carried out to evaluate the treated fish’s interaction behaviors toward their conspecific fish. From the results, chronic waterborne exposure of both chemicals did not statistically change the fish interaction toward their conspecifics, which was indicated by no statistical differences found in conspecific interaction time percentage, longest conspecific interaction time percentage, and average distance to the conspecific’s separator for the social interaction test (Figure S1).

### 3.3. Chronic Exposure to Apalutamide Slightly Reduced the Fear Response of Zebrafish

Afterward, to evaluate whether the exposure of both AR blockers and DHT could alter the fear response of zebrafish, the fear response assay was conducted by using a convict cichlid as the stimulus to induce predator avoidance response in the tested zebrafish. Based on the results, APA-treated fish were found to have a slightly less pronounced fear response toward the convict cichlid. This abnormality was signified by the statistically higher approaching predator time percentage than the untreated group, although no statistical differences were found in another behavior endpoint (Figure 3C,D and Figure S2B). Meanwhile, chronic exposure to ENZ and DHT was not able to alter the fear response of the zebrafish, as shown by the relatively similar levels of both endpoints to those of the vehicle control group (Figure 3C,D).

### 3.4. Chronic Exposure to Enzalutamide Resulted in a Tightened Shoal Formation

Next, the capability of the fish to form a shoal was also evaluated by conducting the shoaling assay. Interestingly, chronic exposure to ENZ at 1 ppm caused the fish to swim in a relatively tight shoal, as indicated by the statistically lower average farthest neighbor distance (Figure 3H and Figure S2C). On the contrary, APA and DHT seemed to have no robust effect in altering the shoaling behaviors in zebrafish since all of the shoaling behaviors-related endpoints did not show any statistical differences from the control group (Figure 3E–H).

### 3.5. PCA and Heatmap Clustering Analysis of the Observed Behavior Alterations After Exposure to Enzalutamide, Apalutamide, and Dihydrotestosterone

**Figure 4:**
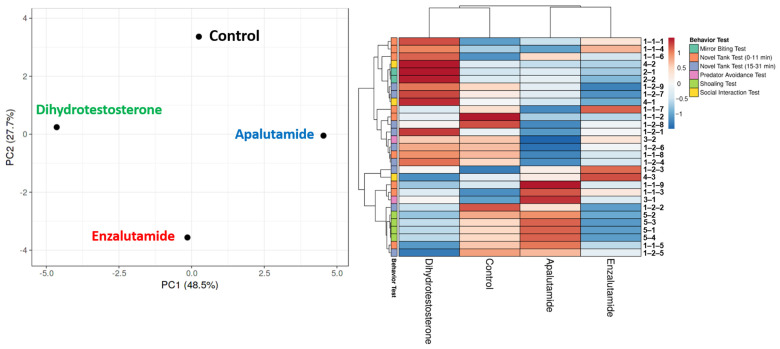
Principal component analysis (**left**) and hierarchical clustering analysis (**right**) of multiple behavior activity endpoints in zebrafish after being exposed either to DMSO (vehicle control), enzalutamide, apalutamide, or dihydrotestosterone. The color bar beside the heatmap clustering showed the range of the behavioral endpoint value after data calculation. Low values tended towards a blue color, while the higher values tended towards a red color.

Considering the number of behavior endpoints that were calculated in the current study, PCA and heatmap clustering analysis were conducted to reduce the dimensionality of the high-dimensional data of the calculated behavior endpoints, which might help in comprehending the behavior differences between every tested group. From the results, both AR blockers were grouped in the same cluster, highlighting their similar behavior effects toward zebrafish, with the most different heatmap pattern in the shoaling-related behavior endpoints (Figure 4). Meanwhile, the DHT-treated group was found to be grouped in the same cluster as the vehicle control group, indicating that the behavior alterations caused by DHT might not be as severe as the tested AR blockers, with the most evident differences between DHT-treated and control groups, which can be observed in the locomotion- and aggression-related behavior endpoints (Figure 4).

### 3.6. The Binding Energy of Enzalutamide, Apalutamide, and DHT to AR by Molecular Docking Assay

From the molecular docking results, in terms of ARs in complex with ENZ, the most ideal binding conformation had a binding energy of −6.6 kcal/mol with amino acid interactions such as conventional hydrogen bonding with ASN727, ASN833, and ARG840; carbon–hydrogen bonding with PHE826; halogen–fluoride interaction with GLU829 and GLU837; pi–cation interaction with GLU837 and ARG840; alkyl interaction with LEU728, LEU830 and LYS836; and lastly, pi–alkyl interaction with LEU830. Meanwhile, for ARs in complex with APA, the most ideal binding conformation had a binding energy of −7 kcal/mol with amino acid interactions such as conventional hydrogen bonding with ASN727, ASN833, and ARG840; halogen (fluorine) interaction with GLY724 and GLU829; pi–anion interaction with GLU829 and GLU837; alkyl interaction with PRO723, LEU830, and LYS836; pi–alkyl interaction with only PHE673, while an unfavorable bump was also discovered in the nitrogen aromatic ring of the ligand. On the other hand, the most ideal binding conformation for ARs in complex with DHT had a binding energy of −6.3 kcal/mol with amino acid interactions such as a conventional hydrogen bond with PHE673 and an alkyl bond with PRO723 and LEU830. Finally, for ARs in complex with tiratricol, the most ideal binding conformation had a binding energy of −5.3 kcal/mol with amino acid interactions such as van der Waals bonding with GLY724; conventional hydrogen bonding with GLU829 and ASN833; pi–anion bond with GLU837; amide–pi stacked interaction with PRO723; alkyl bonding with PRO671 and PRO672; and pi–alkyl bonding with PHE673 and TYR834. In summary, the results showed the potential of the compounds as inhibitors, as they exhibited a negative binding effectivity that is relatively high according to the standard of drugs (−6.00 kcal/mol) [40]. The more negative or higher the binding energy, the more stable the complex, thus proving potential effectiveness (Table 2).

## 4. Discussions

The present study confirms the behavioral alterations in adult male zebrafish caused by chronic exposure to AR blockers, ENZ and APA, and DHT, an AR activator. As expected, both AR blockers and DHT exposures resulted in different behavioral abnormalities in the fish, as indicated by the PCA and heatmap clustering results that showed a relatively different pattern of values between each group. In addition, based on the molecular docking results, all of the tested chemicals also showed relatively higher binding energy to AR. While it was tested in human AR, these results might still indicate that these ligands exhibit high binding affinity to zebrafish AR, considering that the zebrafish gene encodes the true orthologue of the human *ar* gene, suggesting strong potential for biological activity via AR-mediated pathways and potentially disrupting androgen-dependent processes like reproduction, development, or behavior, as shown in the current results. In addition, since ENZ and APA have higher binding energy, they may outcompete endogenous androgens like DHT for AR binding, potentially suppressing normal androgenic signaling in zebrafish.

Here, chronic exposure to ENZ at a relatively low concentration resulted in a slightly lower locomotor activity during the novel tank test and a tighter shoaling formation in the shoaling test. The novel tank test is a behavioral analysis in zebrafish that evaluates two important aspects of fish behavior responses toward a novel environment, which were locomotion, which reflects the fish’s spontaneous activity, and their vertical exploratory activity, based on the tendency of this species to initially dive to the bottom of the tank with a gradual swim to the upper areas of the tank [41]. The decreased locomotor activity in ENZ-treated fish might indicate high anxiety or behavioral inhibition possessed by the fish [21]. Interestingly, a similar phenomenon was observed in a previous study in mice. From their study, it was found that exposure to ENZ in aged castrated mice reduced spontaneous activity in the open field test, a similar assay to a novel test that is used for rodents, highlighting the possibility of ENZ in evoking depression-like behavior in zebrafish [42,43]. Generally, this typical behavior in zebrafish is indicated by hypolocomotion that resembles motor retardation and disrupted shoaling that indicates social withdrawal symptoms, as also observed in the ENZ-treated fish group [43,44]. Shoaling is the aggregation of individual zebrafish to form a tight group in mixed-gender groups with structured hierarchies that are influenced by their social experiences [45,46]. Changes in shoaling, such as tightening of the shoal, can be evoked by various factors, including predator or alarm pheromone exposure and chronic stress [46,47]. Thus, the current results implied the consistent results of ENZ effects in causing higher levels of stress in zebrafish, as indicated by the disruption in their locomotion and shoaling behavior.

Meanwhile, slightly different behavioral alterations were also observed in zebrafish after exposure to APA. To the best of our knowledge, there were only a few studies that addressed the toxicity of APA, especially in fish behaviors. In humans, a prior cohort study of the therapeutic use of second-generation anti-androgens, including APA, in men demonstrated that these drugs had a large and clinically significant increased risk of depression [48]. Similar to ENZ, the slightly altered locomotor activity observed in the APA-treated fish might be due to the depression-like behaviors that were elicited by APA. In addition, this second-generation AR inhibitor has also been associated with CNS-related disorders in men with prostate cancer, including mental impairment disorders and fatigue, which might explain the observed compromised locomotion in the exposed fish, although it was not as severe as ENZ [49]. Furthermore, changes in the fear response were also observed in the APA-treated fish. In general, fish have been well-known to possess some capacity to consciously experience fear in forms of cognitive, neurophysiological, and behavioral features of fear responses [50]. APA has been reported to be potentially able to penetrate the blood–brain barrier, causing CNS-related adverse effects, as supported by a prior study in humans that found a numerically increased odds of neurocognitive impairment in men who took hormone therapy, which might also be the reason for the observed alteration in zebrafish’s fear response [49,51]. Furthermore, the divergence in fear response profiles between the ENZ and APA groups is particularly intriguing given their shared mechanism as AR antagonists. This discrepancy may be attributed to differential off-target effects. For instance, enzalutamide is known to have AR-independent effects, such as the ability to inhibit GABA receptors in the brain that play a key role in the development of anxiety and various stages of fear memory, which include formation, consolidation, retention, reconsolidation, and extinction [52,53,54]. While APA can also act as a weak antagonist at the GABA_A_ receptor, comparative pharmacokinetic studies indicate that APA maintains lower steady-state brain levels compared to ENZ due to distinct blood–brain barrier penetration properties [13]. Therefore, this suggests that, despite equivalent waterborne exposure concentrations (1 ppm), the internal effective dose of APA within the neural circuits regulating fear (e.g., the medial dorsal pallium) may be lower than that of ENZ, resulting in a significantly attenuated fear response in the APA-treated fish compared to the ENZ group. Given the sensitivity of zebrafish fear-related behaviors to stress-axis dysregulation, this finding highlights the potential complexity of androgen antagonist actions on neural circuits governing defensive behavior, and future studies utilizing specific GABA_A_ agonists/antagonists would be required to disentangle these overlapping pathways. In addition, the Principal Component Analysis (PCA) and heatmap clustering (Figure 4) provide a holistic view of the behavioral alterations induced by AR antagonists. While ENZ and APA are grouped together—reflecting their shared identity as AR blockers distinct from the AR activator DHT—they exhibit a clear spatial separation within their cluster. This divergence is driven by specific behavioral endpoints where the two compounds elicit non-identical phenotypes. Specifically, the shoaling behavior endpoints contributed most heavily to this separation; ENZ induced a robust ‘tightening’ of the shoal (a defensive, anxiety-like response), whereas APA-treated fish maintained normal inter-fish distances as mentioned above. Furthermore, the fear response vector highlights a unique attenuation of predator avoidance in the APA group, contrasting with the response observed in ENZ-treated fish. These multivariate differences support the hypothesis that while both compounds block the androgen receptor, their neurobehavioral toxicity profiles are distinct. This is likely attributable to the differential blood–brain barrier penetration and GABA_A_ receptor affinity described earlier, which results in a more severe anxiogenic and motor-suppressive phenotype for ENZ compared to the relatively milder behavioral footprint of APA [13]. Taken together, the findings of the present study provide evidence on the adverse effects of ENZ and APA on male zebrafish behaviors, supporting the potential ability of these drugs to compromise CNS function.

On the other hand, different results were shown by the DHT-treated fish. While both AR blockers caused slightly decreased locomotor activity in male zebrafish, exposure to DHT resulted in higher locomotion. This result is in line with the previous study in male rats that found that treatment with testosterone increased their locomotion [55]. While their study eventually concluded that testosterone had an anxiolytic effect in male rats, it might not be the case in the current study, since generally, besides an increase in locomotor activity, a reduction in anxiety in zebrafish behaviors is also indicated by enhanced exploration of the upper zone, as shown in the previous study [56,57]. Instead, the current findings might indicate anxiogenic effects of DHT, as bottom-dwelling and hyperactivity, as well as erratic movements, have been suggested as indicators of anxiety in the ‘open tank’ paradigm in fish [58]. In addition, as expected, a statistical increase in aggression was observed in the DHT-treated group. The effect of DHT in enhancing animals’ aggression, especially mammals, has been well addressed in previous studies. For instance, DHT elicited significantly higher levels of aggressive responses to intact intruders in the castrated male hamsters [59]. Aside from affecting male behaviors, androgens have also been demonstrated to induce aggression in females. In female rats, dihydrotestosterone propionate (DHTP) was found to activate their aggressive response as well as their sexual behavior, which was similar to the results shown in another study that used ovariectomized guinea pigs [30,60]. On the other hand, the functions of DHT in teleost fish have been largely unexplored despite all components necessary for DHT biosynthesis and function being present and evolutionarily conserved in teleost fish, and moreover, it is generally assumed that DHT lacks a significant physiological role [61]. This might be due to the most biologically active androgen in teleost fish being 11-ketotestosterone (KT), a non-aromatizable androgen, despite their possession of testosterone and DHT, unlike mammals and birds, where testosterone and DHT are the primary androgens [62]. Nonetheless, several studies have evaluated the effect of DHT in fish; however, different results were observed. While studies on African catfish and Japanese eel have shown that DHT has no effect on spermatogenesis [63,64], another study demonstrated an androgenic potency of DHT in plasma of fathead minnow (*Pimephales promelas*) [61], which seemed to be consistent with the current findings, suggesting a species-specific response that presumably associated with differences in androgen receptor (AR) properties, particularly AR–steroid binding affinity as in several teleost species, including zebrafish, DHT has demonstrated a higher binding affinity for the AR compared to KT [65].

The current findings highlight the importance of regulating the waste discharge management of this endocrine and its blockers to the environment, especially aquatic environments, after several studies found various organic pollutants with similar activities to the tested AR blockers. For instance, a study found antagonist activities for the androgen receptor in river water extracts in Taiwan [66]. Similarly, multiple hormonal activities, including androgenic and antiandrogenic activities, were also reported in surface water and sediment from the Pearl River system in South China, as well as in the river in several European countries [67,68,69,70]. Accordingly, the behavioral alterations demonstrated in the present study may also manifest in natural aquatic environments as a consequence of exposure to endocrine-disrupting chemicals (EDCs), which enter these ecosystems primarily through effluent discharges from wastewater treatment facilities and diffuse runoff from terrestrial sources, and such contaminants have the potential to affect a broad spectrum of aquatic organisms. Generally, changes in behavior that affect individual fitness—such as survival, growth, or reproductive success—or alter spatial and temporal distribution can ultimately influence population dynamics, including changes in population size, structure, and spatiotemporal distribution [71]. For example, the alteration that was observed in the fish after exposure to APA could lead to a shift in predator–prey interactions and eventually restructure food webs [72]. Similarly, the inability to shoal effectively, as indicated in the ENZ-treated fish, can also make prey more vulnerable to predation [73]. Moreover, their effects on fish swimming performance could also compromise the ability to escape predators, while hyperactivity-like behaviors that were observed after DHT exposure may increase conspicuousness to predators [74]. Meanwhile, the enhanced aggression caused by DHT exposure may have consequences for social hierarchies and breeding outcomes of individuals [75]. Taken together, given the widespread occurrence of neurotoxic EDCs in aquatic environments and their demonstrated capacity to disrupt essential behaviors in fish, a combination of improved wastewater treatment, stricter regulation, green chemistry initiatives, and behavior-based ecological risk assessments is essential to mitigate their ecological impact and protect aquatic biodiversity [76].

## 5. Conclusions

In conclusion, we show for the first time that chronic exposure to two AR blockers, ENZ and APA, resulted in behavior alterations in male zebrafish, including disruptions in locomotor activity, shoaling formation, and fear response. Meanwhile, the present study also demonstrated DHT in affecting the physiological role of zebrafish, as indicated by higher locomotion and aggressiveness levels as well as altered exploratory behaviors, signifying its androgenic potency. Nevertheless, ones have to keep in mind that all behavioral assays were conducted using the same test tank and followed a fixed testing sequence, which may introduce potential order effects and environmental habituation. Although tests were distributed across multiple days, rest periods were provided between assays, and habituation procedures were applied prior to each test (except for the novel tank test), subtle carryover effects cannot be completely excluded. However, as all experimental groups were subjected to identical testing conditions and sequences, these factors are unlikely to differentially affect treatment groups. Future studies may further address this limitation by counterbalancing test order or using distinct testing environments for individual behavioral assays. Finally, the molecular docking results indicate that ENZ, APA, and DHT exhibit high binding affinity to the zebrafish androgen receptor, with ENZ and APA displaying relatively stronger interactions than the natural ligand DHT, which might suggest that both AR antagonists functionally interfere with androgen receptor signaling in zebrafish, thereby disrupting androgen-mediated physiological and behavioral processes. However, although the present study integrates behavioral assessments with molecular docking analyses to infer androgen receptor (AR) involvement, direct experimental validation of AR pathway modulation was not conducted. Specifically, changes in AR target gene expression or AR-associated protein activity in neural tissues were not measured. Such analyses would provide stronger mechanistic evidence linking receptor-level interactions to the observed behavioral phenotypes. Future studies incorporating transcriptomic, proteomic, or immunohistochemical approaches targeting AR signaling pathways in behaviorally relevant brain regions will be essential to further elucidate the mechanistic basis of androgen receptor-mediated behavioral modulation in adult zebrafish. Furthermore, the strong binding supports structural conservation of the AR ligand-binding domain between zebrafish and humans, reinforcing the zebrafish model’s relevance in endocrine disruption studies. Given their presence in the environment, these compounds may pose ecotoxicological risks to aquatic organisms. However, future studies should be conducted to investigate the underlying neural mechanisms by which AR blockers or DHT affect fish behaviors, particularly at the molecular and signaling pathway levels. Additionally, research could explore the long-term consequences of AR blockade on male zebrafish physiology, as might be indicated in courtship behavior and reproductive fitness, to assess the role of androgen signaling in regulating social interactions and reproduction. The results of this study will contribute to a deeper understanding of the relationship between androgens and behaviors in vertebrates, establishing a foundation for further research on the physiological and neurological mechanisms underlying androgen-mediated behaviors.

## Figures and Tables

**Table 1:** Grid size used for reverse docking of androgen receptor.

	X	Y	Z
Center (Å)	15.651	17.306	26.711
Dimensions (Å)	23.686	12.569	14.894

**Table 2:** The binding energy of the androgen receptor–ligand complexes.

Protein	Ligand	Binding Energy (kcal/mol)
Androgen Receptor	Enzalutamide	−6.6
	Apalutamide	−7.0
	Tiratricol	−6.3
	Dihydrotestosterone	−6.3

## Data Availability

The data that support the findings of this study are available from the corresponding author upon reasonable request.

## Supplementary Materials

The following supporting information can be downloaded at: https://www.mdpi.com/article/10.3390/biology15050393/s1, Figure S1: Conspecific social interaction (**(A)** Conspecific interaction time percentage, **(B)** average distance to the conspecific’s separator, and **(C)** longest conspecific interaction percentage) behavior endpoints of zebrafish after being systemically exposed either to 1 ppm of enzalutamide (ENZ) (red), apalutamide (APA) (blue), or dihydrotestosterone (DHT) (green), compared to vehicle control (black). The data are expressed as the median with an interquartile range. The statistical analyses were conducted by the Kruskal-Wallis test followed by Dunn’s multiple comparisons test (*n* = 30); Figure S2: Comparison of representative trajectories of single or multiple fish between control and (A) DHT-treated fish in the mirror biting test, (B) APA-treated fish in the fear response test, and (C) ENZ-treated fish in the shoaling test; Table S1: Summary of several studies that addressed the behavioral effects of enzalutamide, apalutamide, or dihydrotestosterone and its metabolic relatives in various organisms; Table S2: Summary of fish behavioral endpoints measured in each behavior test; Table S3: Summary of Two-way ANOVA and Dunnett’s multiple comparisons tests results between the control and every treatment group of each behavior endpoint from the novel tank test. References [77,78,79,80,81,82,83,84] are cited in the Supplementary Materials.
