# Supplementary material for: Behavioral Alterations in Male Zebrafish After Administration of Androgen Receptor Blockers and an Activator
Source: Biology (Basel). 2026 Feb 27;15(5):393. doi: 10.3390/biology15050393 (PMC12984835; doi:10.3390/biology15050393)
Supplement: Supplementary file 1 [file biology-15-00393-s001.zip › biology-4166793-supplementary.pdf]

## Supplemental Files

**Table S1.** Summary of several studies that addressed the behavioral effects of enzalutamide, apalutamide, or dihydrotestosterone and its metabolic relatives in various organisms.

| Androgen Receptor Ligands | *In vivo* Models | Behavior Results | References |
|---|---|---|---|
| Enzalutamide | Humans | Fatigue and hypertension were the most common clinically relevant adverse events associated with enzalutamide treatment | [77] |
| | | Fatigue, falls, and fractures were more common in patients who received enzalutamide than in those who received a placebo | [78] |
| | | Caused fatigue, back pain, hot flush, hypertension, diarrhoea, weight decrease, and pain in the extremities, as the most common adverse events | [79] |
| | Aged castrated mouse | Reduced spontaneous activity and exploratory behavior | [17] |
| | Aged castrated mice | Reduced spontaneous activity and increased depressive-like behaviors | [43] |
| Apalutamide | Humans | Associated with higher rates of fatigue and falls than placebo | [80] |
| | | Resulted in fatigue as a very common adverse effect | [81] |
| Second-generation androgen receptor inhibitors | Humans | Increased risk of depression | [48] |
| | | Fatigue and mental impairment disorders | [49] |
| Dihydrotestosterone | Castrated male hamsters | Elicited significantly higher levels of aggression | [59] |
| | Male lizard (*Anolis carolinensis*) | Increased courtship behavior in combination with 17β-estradiol | [82] |
| | Female mice | Injections on the day of birth caused mice to fight sooner in adult life | [83] |
| | Male Long–Evans rats | Induce place preferences | [84] |
| Testosterone (precursor to DHT) | Wistar rats | Increased locomotion | [55] |
| Dihydrotestosterone propionate (a long-acting form of DHT) | Female rats | Synergistically activate aggression with estradiol benzoate (EB) | [30] |
| | Ovariectomized guinea pigs | Significantly influences aggression with the concurrent administration of EB | [60] |

**Table S2.** Summary of fish behavioral endpoints measured in each behavior test.

| Index | Behavior endpoints (units) | Definition | Applied to |
|---|---|---|---|
| 1–1–1 & 1–2–1 | Average speed (cm $s^{-1}$) | Total distance traveled by fish divided by total time duration | Novel Tank Test |
| 1–1–2 & 1–2–2 | Freezing movement time ratio (%) | Total percentage of time when the fish's speed is less than 1 cm $s^{-1}$ | |
| 1–1–3 & 1–2–3 | Swimming movement time ratio (%) | Total percentage of time when the fish's speed is between 1 and 10 cm $s^{-1}$ | |
| 1–1–4 & 1–2–4 | Rapid movement time ratio (%) | Total percentage of time when the fish's speed is more than 10 cm $s^{-1}$ | |
| 1–1–5 & 1–2–5 | Time in top duration (%) | Total time spent in the top portion of the novel tank in percentage | |
| 1–1–6 & 1–2–6 | Number of entries to the top | The total time fish enter the upper half of the tank | |
| 1–1–7 & 1–2–7 | Latency to enter the top (s) | The amount of time it takes the fish to cross into the upper half of the tank | |
| 1–1–8 & 1–2–8 | Total distance traveled in the top (cm) | Total distance traveled in the top portion of the novel tank | |
| 1–1–9 & 1–2–9 | Thigmotaxis (cm) | The average distance of the fish from the center of the tank | |
| 2–1 | Mirror biting time percentage (%) | Total percentage of time when the fish stayed in the mirror biting zone | Mirror Biting Test |
| 2–2 | Longest duration in the mirror side percentage (%) | Total percentage of fish longest duration stayed in the mirror biting zone | |

| | | | |
|---|---|---|---|
| 3–1 | Approaching predator time percentage (%) | Total percentage of time when fish stayed in the approaching predator zone | Fear Response Test |
| 3–2 | Average distance to the predator's separator (cm) | Average distance of fish to the predator's separator | |
| 4–1 | Conspecific interaction time percentage (%) | Total percentage of time when fish stayed in the conspecific interaction zone | Conspecific Social Interaction Test |
| 4–2 | Average distance to the conspecific's separator (cm) | Average distance of fish to the conspecific's separator | |
| 4–3 | Longest conspecific interaction percentage (%) | The total percentage of fish longest duration stayed in the conspecific interaction | |
| 5–1 | Average inter-fish distance (cm) | The average distance between the body center of every member of the shoal | Shoaling Test |
| 5–2 | Average shoal area ($cm^2$) | Average size of the shoal | |
| 5–3 | Average nearest neighbor distance (cm) | Distance from the body center of each fish to the closest neighboring fish | |
| 5–4 | Average farthest neighbor distance (cm) | Distance from the body center of each fish to the farthest neighboring fish | |

**Table S3.** Summary of Two-way ANOVA and Dunnett's multiple comparisons tests results between the control and every treatment group of each behavior endpoint from the novel tank test (ns = not statistically significant).

| | Two-way ANOVA Results | | | Dunnett's multiple comparisons test | | |
|---|---|---|---|---|---|---|
| **Behavior Endpoints** | **Source of Variation** | **F (DFn, DFd)** | ***P* Value** | **Test details** | **Summary** | ***P* Value** |
| Average Speed | Interaction | F (18, 696) = 1.801 | 0.0217 | Control vs. Enzalutamide | ns | 0.5730 |
| | Time (Row Factor) | F (4.693, 544.4) = 17.68 | <0.0001 | Control vs. Apalutamide | ns | 0.4729 |
| | Group (Column Factor) | F (3, 116) = 2.488 | 0.0640 | Control vs. Androstan | *** | 0.0002 |
| Freezing Movement Time Ratio | Interaction | F (18, 696) = 1.668 | 0.0402 | Control vs. Enzalutamide | *** | 0.0003 |
| | Time (Row Factor) | F (4.630, 537.1) = 7.398 | <0.0001 | Control vs. Apalutamide | ns | 0.0855 |
| | Group (Column Factor) | F (3, 116) = 1.734 | 0.1638 | Control vs. Androstan | ** | 0.0088 |
| Swimming Movement Time Ratio | Interaction | F (18, 696) = 1.954 | 0.0103 | Control vs. Enzalutamide | **** | <0.0001 |
| | Time (Row Factor) | F (4.775, 553.9) = 2.655 | 0.0239 | Control vs. Apalutamide | ** | 0.0022 |
| | Group (Column Factor) | F (3, 116) = 2.270 | 0.0841 | Control vs. Androstan | ns | 0.0579 |
| Rapid Movement Time Ratio | Interaction | F (18, 696) = 1.859 | 0.0165 | Control vs. Enzalutamide | ns | 0.9602 |
| | Time (Row Factor) | F (4.672, 541.9) = 8.998 | <0.0001 | Control vs. Apalutamide | ** | 0.0016 |
| | Group (Column Factor) | F (3, 116) = 2.854 | 0.0403 | Control vs. Androstan | ns | 0.0661 |
| Time in Top Duration | Interaction | F (18, 696) = 1.125 | 0.3219 | Control vs. Enzalutamide | ns | 0.1507 |
| | Time (Row Factor) | F (4.829, 560.2) = 29.61 | <0.0001 | Control vs. Apalutamide | ns | >0.9999 |
| | Group (Column Factor) | F (3, 116) = 2.138 | 0.0992 | Control vs. Androstan | ** | 0.0013 |
| Number of Entries to The Top | Interaction | F (18, 696) = 1.672 | 0.0395 | Control vs. Enzalutamide | ns | 0.8734 |
| | Time (Row Factor) | F (5.339, 619.3) = 7.889 | <0.0001 | Control vs. Apalutamide | ns | 0.1693 |
| | Group (Column Factor) | F (3, 116) = 0.8299 | 0.4800 | Control vs. Androstan | ns | 0.7514 |
| Latency to Enter The Top | Interaction | F (18, 696) = 1.353 | 0.1482 | Control vs. Enzalutamide | ns | 0.9330 |
| | Time (Row Factor) | F (4.465, 518.0) = 34.89 | <0.0001 | Control vs. Apalutamide | ns | 0.4036 |
| | Group (Column Factor) | F (3, 116) = 0.5376 | 0.6574 | Control vs. Androstan | ns | 0.9822 |
| Total Distance Traveled in The Top | Interaction | F (18, 696) = 0.8027 | 0.6983 | Control vs. Enzalutamide | ns | 0.6275 |
| | Time (Row Factor) | F (4.872, 565.1) = 16.67 | <0.0001 | Control vs. Apalutamide | ns | 0.1014 |
| | Group (Column Factor) | F (3, 116) = 0.3987 | 0.7542 | Control vs. Androstan | ns | 0.7663 |
| | Interaction | F (18, 696) = 1.004 | 0.4532 | Control vs. Enzalutamide | ns | 0.5939 |
| | Time (Row Factor) | F (5.475, 635.1) = 10.79 | <0.0001 | Control vs. Apalutamide | ns | 0.9143 |

| | | | | | | |
|---|---|---|---|---|---|---|
| Average Distance to Center of the Tank | Group (Column Factor) | F (3, 116) = 0.4670 | 0.7058 | Control vs. Androstan | ns | 0.9951 |

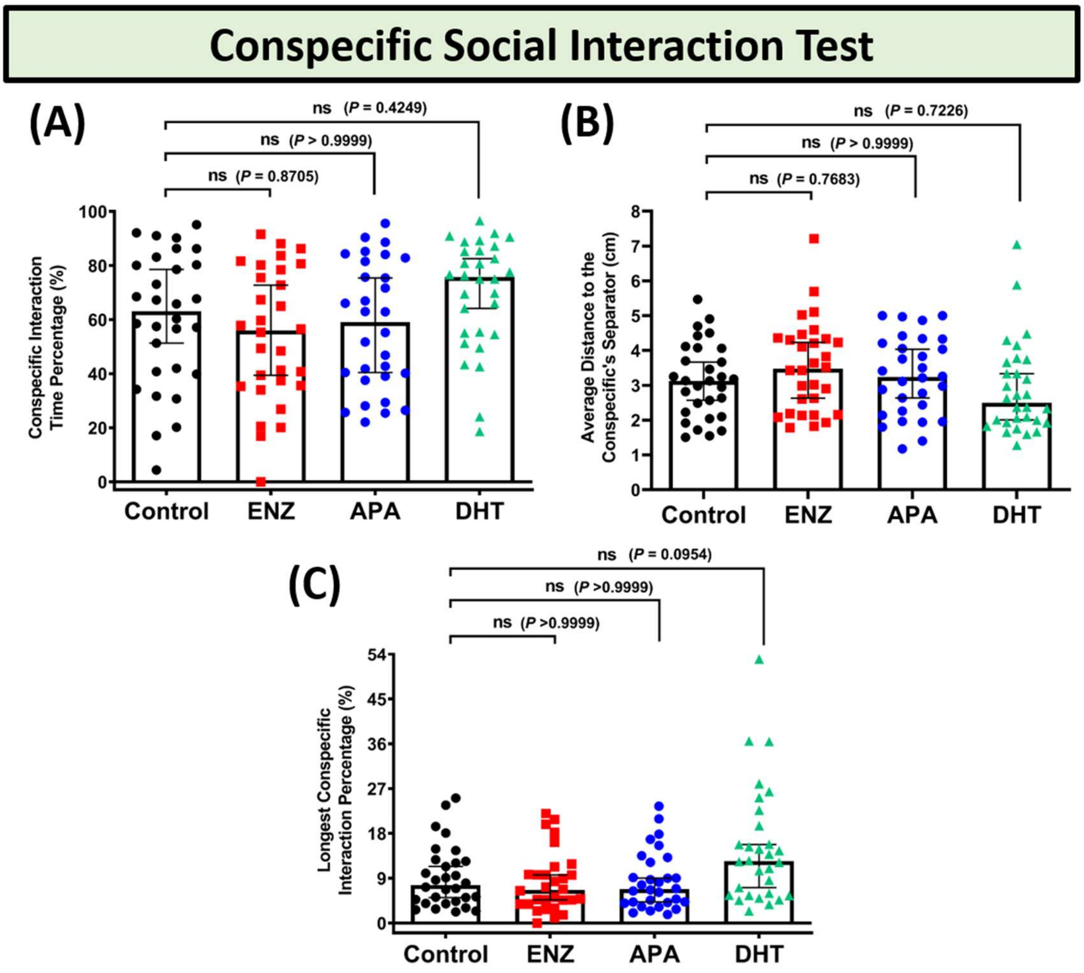


**Figure S1.** Conspecific social interaction (**(A)** Conspecific interaction time percentage, **(B)** average distance to the conspecific's separator, and **(C)** longest conspecific interaction percentage) behavior endpoints of zebrafish after being systemically exposed either to 1 ppm of enzalutamide (ENZ) (red), apalutamide (APA) (blue), or dihydrotestosterone (DHT) (green), compared to vehicle control (black). The data are expressed as the median with an interquartile range. The statistical analyses were conducted by the Kruskal-Wallis test followed by Dunn's multiple comparisons test ($n$ = 30).

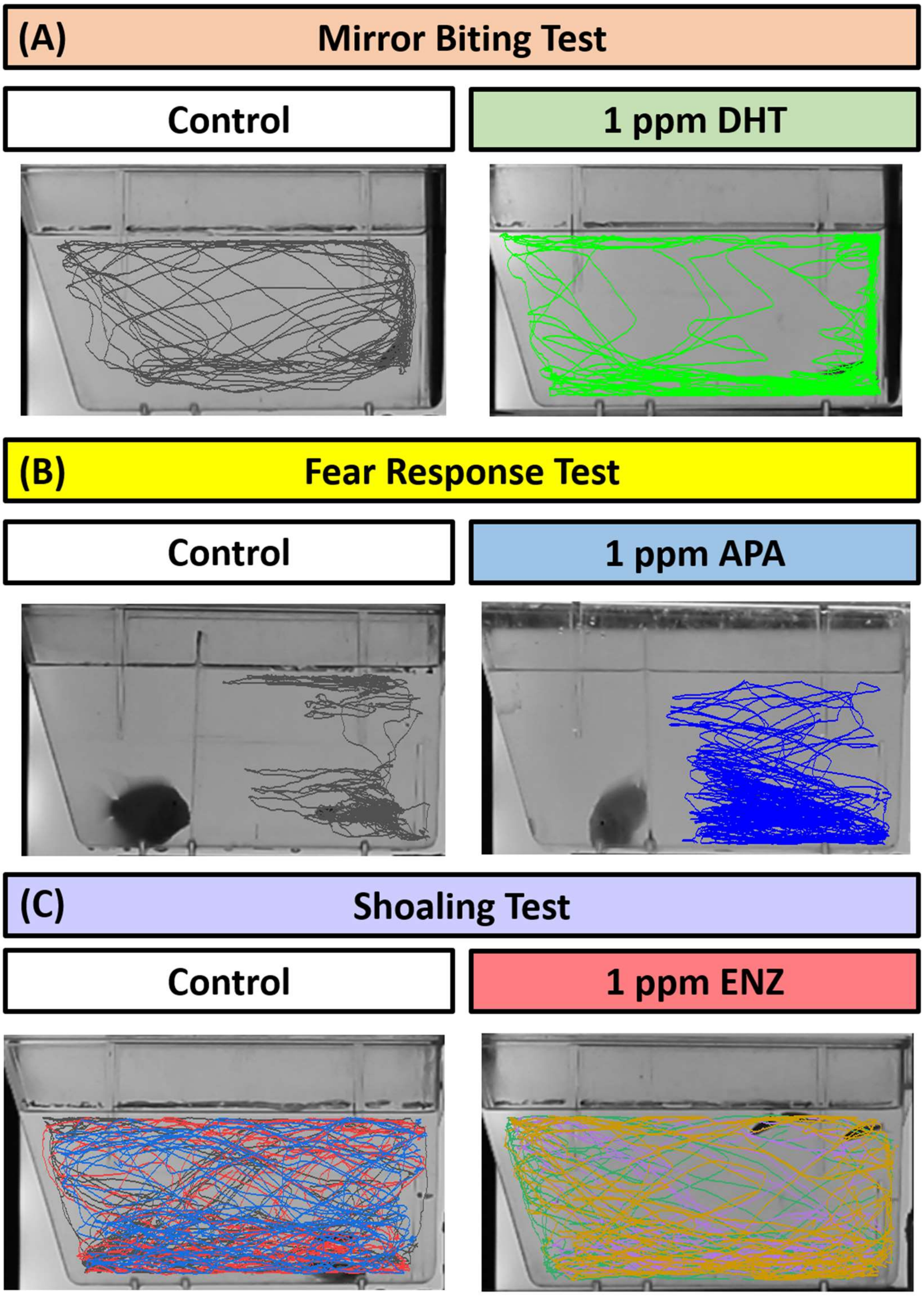


**Figure S2.** Comparison of representative trajectories of single or multiple fish between control and **(A)** DHT-treated fish in the mirror biting test, **(B)** APA-treated fish in the fear response test, and **(C)** ENZ-treated fish in the shoaling test.
